# Solution of an elusive pigment crystal structure from a thin film: a combined X-ray diffraction and computational study†
†Electronic supplementary information (ESI) available: AFM micrographs of ∼7 nm thick samples; XRR data of ∼7 nm thick samples prepared at substrate temperatures of 200 and 350 K, including data fits and electron density profiles; chemical structure diagrams of the 2F-EPI and 2Cl-EPI derivatives; overlays of the thin film EPI structure with the halogenated derivative structures; graphical representation of the contributions of different intermolecular interactions to the Hirshfeld surfaces presented in Fig. 6. Crystallographic information file (cif) for the structure of EPI solved from a thin film, CCDC 1528520. For ESI and crystallographic data in CIF or other electronic format see DOI: 10.1039/c7ce00227k



**DOI:** 10.1039/c7ce00227k

**Published:** 2017-03-14

**Authors:** Andrew O. F. Jones, Christian Röthel, Roman Lassnig, O. N. Bedoya-Martínez, Paul Christian, Ingo Salzmann, Birgit Kunert, Adolf Winkler, Roland Resel

**Affiliations:** a Institute of Solid State Physics , Graz University of Technology , Petersgasse 16 , 8010 Graz , Austria . Email: andrew.jones@tugraz.at; b BioTechMed-Graz , Austria; c Department of Pharmaceutical Technology , Institute for Pharmaceutical Sciences , Karl-Franzens University of Graz , Universitätsplatz 1 , 8010 Graz , Austria; d Institut für Physik , Humboldt-Universität zu Berlin , Brook-Taylor Straße 6 , 12489 Berlin , Germany

## Abstract

Epindolidione, a hydrogen-bonded derivative of the organic semiconductor tetracene, is an organic pigment which has previously been used to produce stable OFETs with relatively high hole mobilities. Despite its use as an inkjet pigment and organic semiconductor, the crystal structure of epindolidione has proved elusive and is currently unknown. In this work, we report a crystal structure solution of epindolidione determined from vapor deposited thin films using a combined experimental and theoretical approach. The structure is found to be similar to one of the previously reported epindolidione derivatives and is most likely a surface-mediated polymorph, with a slightly different crystal packing compared to the bulk powder. The effect of substrate temperature on film morphology and structure is also investigated, where it is found that the crystallite orientation can be tuned by deposition at different substrate temperatures. The results also illustrate the possibilities for crystal structures to be solved from thin films.

## Abstract 2

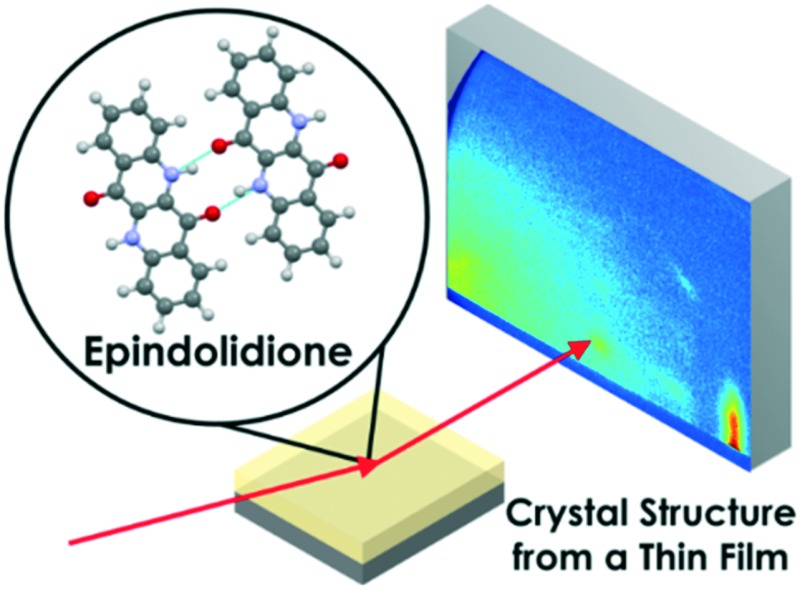
The previously unknown crystal structure of epindolidione has been determined from a thin film by combining diffraction data with calculations.

## Introduction

Hydrogen-bonded pigments are extremely stable, generally non-toxic materials which have recently found applications in organic electronics due to the observation of promising charge carrier mobilities in organic field-effect transistors (OFETs).^1–6^ Of the hydrogen-bonded pigments studied to date, epindolidione (EPI), a hydrogen-bonded derivative of the well-known organic semiconductor tetracene, is one of the most promising, with hole mobilities of up to 1.5 cm^2^ V^–1^ s^–1^ observed in thin film transistors.^3^ As for any molecular material, the physical properties are strongly influenced by the arrangement of the molecules in the solid state (*i.e.* the crystal structure).^7^ For charge transport in thin films, the molecular arrangement in the vicinity of the substrate is particularly important,^8^ and it has also been shown that new polymorphs, known as surface-mediated phases, can appear in this region.^9^ Structural characterization of thin films of materials to be used in organic electronic devices is therefore crucial to the understanding of their behavior.

In thin films of EPI, it was previously reported that the substrate temperature during film deposition can have a significant impact on the resultant transistor performance.^5^ The strong influence of preparation conditions on film structure is well known, and it was recently shown for another hydrogen-bonded pigment, Tyrian purple, that a new polymorph can be induced at low substrate temperatures.^10^ The performance variation in transistors produced from EPI deposited at different substrate temperatures points to structural differences in the thin films which are yet to be characterized.

EPI films also present an interesting example to study due to the fact that no crystal structure has been reported for this compound; attempts to grow single crystals suitable for structure solution have so far failed (though the crystal structures of two halogenated derivatives were solved).^11^ Such difficulties in the growth of high quality single crystals were also observed for the closely related quinacridone family of hydrogen-bonded pigments.^12^ The structural study of EPI thin films, therefore, presents an opportunity to attempt to solve the crystal structure directly from a thin film, for a material where, to date, no crystal structure has been forthcoming by any other means (such as single crystal X-ray diffraction or powder X-ray diffraction).

However, crystal structure solution from thin films is not a routine technique, and is normally only necessary when a surface-mediated polymorph is present for which single crystals cannot be grown.^9^ There are many difficulties associated with crystal structure solution from a thin film, primarily the indexation of diffraction patterns and the observation of a sufficient amount of data (often only a small number of Bragg peaks are observed). Extraction of reliable peak intensities, normally crucial for structure solution, is also challenging due to the strong influence of crystalline texture on the diffracted intensities.^13–15^ Recently, a method has been developed combining molecular dynamics (MD) simulations with grazing incidence X-ray diffraction (GIXD) data which only requires data sufficient to index and obtain a unit cell; this approach was recently used to solve the thin film structure of the hydrogen-bonded pigment Tyrian purple.^10^ A similar approach to the thin film crystal structure solution of EPI is taken here.

In this work, the growth of vapor deposited films of EPI and the solution of an elusive crystal is described; the impact of substrate temperature during deposition on film structure and morphology is also discussed. The results show the potential for a combination of X-ray diffraction and MD simulations to be used for crystal structure solution from thin films, when even only a very small number of diffraction peaks are observed. The importance of substrate temperature during deposition is highlighted by the observed differences in film structure and molecular orientation on the substrate. Film structure is then linked to the performance of transistors produced from films deposited in a similar manner.

## Experimental & computational methods

Epindolidione (EPI) powder was synthesized following the procedure outlined by Jaffe and Matrick^16^ and taking account of the considerations of Kemp *et al.* ^17^ The powder was sublimation purified before use. EPI films of different thicknesses were deposited by physical vapor deposition onto 1 cm × 1 cm silicon substrates covered with a 150 nm thick layer of thermally grown oxide. Substrates were inserted into a vacuum chamber and cleaned by sputtering with Ar ions before being heated to ∼800 K to further remove contaminants. EPI was deposited from a Knudsen cell source in a vacuum with a base pressure of 2 × 10^–8^ mbar. Film thickness was monitored during deposition using a quartz microbalance where a conversion factor of *Δ* 1 Hz ≈ 0.083 nm was used.^5^ During deposition, the substrate temperature could be set to temperatures in the range of ∼120–800 K (cooling provided by liquid N_2_) and films were deposited at substrate temperatures of 200, 300, and 350 K. A detailed description of the experimental setup and sample mounting used is given elsewhere.^18^

Film morphology was studied by atomic force microscopy (AFM) measurements performed using a Nanosurf Easyscan 2 in tapping mode using PPP-NCLR-50 silicon tips from NANOSENSORS.

Powder X-ray diffraction measurements on bulk EPI powder were performed on a Siemens D500 diffractometer in a Bragg–Brentano configuration. A Cu sealed tube (*λ* = 1.54 Å) was used, with the beam guided through a slit system before the sample; a secondary graphite monochromator was then used in front of a scintillation detector. The simulated powder diffraction pattern for the structure of EPI solved from the thin film was generated using the *Mercury* (CCDC) software package, which was also used for crystal structure visualization.^19^

Specular X-ray diffraction and X-ray reflectivity (XRR) measurements were made on a PANalytical EMPYREAN reflectometer using Cu K_α_ radiation (*λ* = 1.54 Å). On the primary side, a multilayer X-ray mirror was used to generate a parallel beam. On the secondary side, an anti-scatter slit and 0.02 rad Soller slit were used with a PANalytical PIXcel^3D^ detector. Specular X-ray diffraction and XRR data are plotted as a function of *q*_*z*_, the out-of-plane component of the scattering vector, with *q*_*z*_ = 4π/*λ* sin *Θ*, where *λ* is the X-ray wavelength and *Θ* is half of the scattering angle, 2*Θ*. XRR data were fitted and the electron density profile of films determined using the *Stochfit* software package.^20^

Grazing incidence X-ray diffraction (GIXD) experiments were performed at the KMC-2 beamline^21^ at the BESSY II synchrotron radiation source (Helmholtz Zentrum Berlin (HZB), Berlin, Germany) using an X-ray wavelength of 1.00 Å. A 2D VÅNTEC-2000 Mikrogap detector (BRUKER) equipped with an anti-air-scatter cone was used to record intensities. An incident angle of *α*_i_ = 0.13°, close to the critical angle of the substrate, was chosen to enhance the scattered intensities and suppress scattering from the substrate. Reciprocal space maps were calculated from the measured data using the *xrayutilities* library for Python.^22^ Unit cell indexation and the calculation of structure factors was performed using the in-house developed software *PyGID*.^23^

Structure solution using the experimentally determined unit cell constants was performed using a combined molecular dynamics (MD) and density functional theory (DFT) approach. MD simulations were performed using LAMMPS in combination with the CHARMM General Force Field v. 2b7.^24,25^ Several thousand trial structures were generated by randomly placing one molecule into an expanded unit cell (120% the experimental unit cell volume). During the MD simulation run (70 ps with 1 fs time step), the system was allowed to relax while the unit cell shrunk back to the experimentally determined size. After equilibration at the experimental system size and a final energy minimization step, the atomic positions were extracted. Resultant structures were grouped based on their geometric similarity using the Hausdorff metric. In each structural grouping, characteristic features such as distinct hydrogen bond lengths or π–π interaction distances could be identified. Subsequently, to overcome the shortcomings of classical MD simulations, one representative structure from each group was used as an input for DFT geometry optimization as implemented in the VASP program (version 5.4.1).^26–29^ The PBE functional was used for exchange and correlation,^30^ with PAW potentials for all the elements.^31^ The effects of van der Waals interactions (vdW) were included using the pairwise method of Tkatchenko and Scheffler (TS).^32^ A plane-wave cutoff energy of 800 eV was employed. Calculations were performed on the primitive cell, containing 30 atoms, and a 3 × 2 × 1 Monkhorst–Pack *k*-grid to sample the Brillouin zone.^33^ The total energy during the self-consistency loop of each DFT step was converged to 10^–8^ eV. Calculations were performed using the experimental volume, relaxing atomic positions down to a threshold of 10^–2^ eV Å^–1^ on forces. The final optimized geometry was verified by calculating the phonon dispersion using density functional perturbation theory as implemented in VASP.

Hirshfeld surfaces^34,35^ and their corresponding fingerprint plots^36^ for EPI and derivatives were calculated using the Crystal Explorer^37^ software package. Hirshfeld surfaces offer a description of the electron density belonging to a defined unit (in this case molecules). Contrary to other types of molecular surfaces, Hirshfeld surfaces are defined by the molecule and the environment around it; the area enclosed within the surfaces presented here is defined as being where >50% of the electron density belongs to the selected molecule around which the surface is plotted. Two distances are defined for each point on the Hirshfeld isosurface: *d*_e_, the distance to the nearest nucleus outside the surface, and *d*_i_, the distance to the nearest nucleus within the surface. From these values, a normalized contact distance, *d*_norm_, can be defined relative to the van der Waals radii of the atoms involved, where *d*_norm_ is either positive or negative depending on if the intermolecular contact distance is greater or less than the sum of the van der Waals radii.^34^ Plotting *d*_norm_ on the Hirshfeld surface produces a surface with a red/blue/white color scheme; red areas correspond to contact distances shorter than the sum of the van der Waals radii, white areas approximately equal to the sum of the van der Waals radii, and blue areas where the distance is longer than the sum of the van der Waals radii (*i.e.* there are no close contacts). As plotting *d*_norm_ on the Hirshfeld surface requires knowledge of the atom types associated with the values of *d*_e_ and *d*_i_ calculated at each point on the surface, it is possible to define the percentages of interactions to/from the surface arising from specific atom types. In this way, the Hirshfeld surface can be used to get information regarding types of intermolecular interactions occurring with a given molecule for which the surface is plotted.

## Results & discussion

**Fig. 1:**
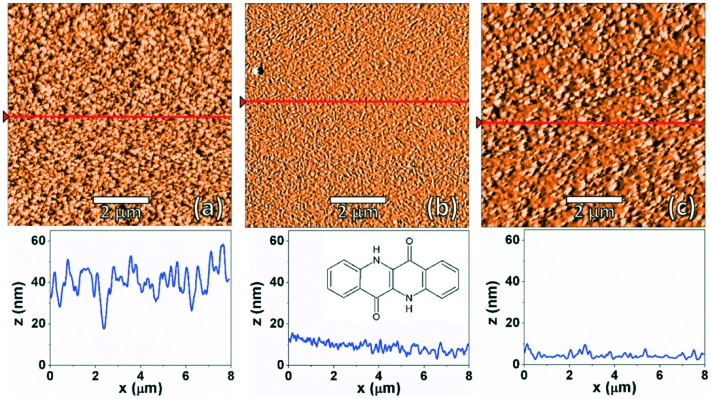
AFM images (top) of epindolidione films with a nominal thickness of 100 nm grown on SiO_2_ surfaces at substrate temperatures of 200 K (a), 300 K (b) and 350 K (c). The red lines correspond to the paths along from which line profiles (bottom) have been generated where the zero value for *z* has been set to the lowest point measured in the AFM image. The chemical structure of epindolidione is given as an inset in the bottom center of the figure.

AFM images were recorded for EPI films deposited at different substrate temperatures to observe any differences in the film morphology. Fig. 1 shows AFM images of nominally ∼100 nm thick films prepared at substrate temperatures of 200, 300 and 350 K. Unless otherwise stated, all results discussed below refer to samples with a thickness of 100 nm. All samples show fully covered substrates with a certain degree of roughness in the multi-domain films. The different substrate temperatures only induce minor differences in the film morphologies. For example, it can be seen that the films prepared at 300 and 350 K are smoother than those prepared at 200 K. Also, a small difference in the island/domain size is also noticeable, with the films prepared at 350 K having larger islands than those prepared at lower temperatures. The observed morphologies are consistent with previous morphological studies of EPI thin films.^5^

**Fig. 2:**
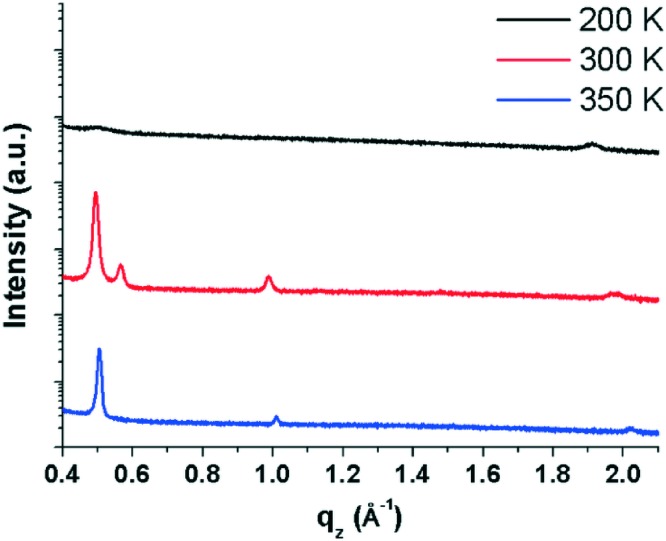
Specular X-ray diffraction patterns of ∼100 nm thick epindolidione films prepared at substrate temperatures of 200, 300 and 350 K showing diffraction peaks arising from lattice planes parallel to the substrate surface. Diffraction patterns are shifted vertically for clarity.

To better understand the molecular arrangement within the thin films, specular X-ray diffraction measurements (probing the periodicities normal to the substrate surface) were performed (Fig. 2). It is immediately apparent from the diffraction patterns that the different substrate temperatures have an impact on the film structure which is not reflected in the morphology, as assessed by AFM. In films prepared at 200 K, two broad, weak Bragg peaks are visible at ∼0.5 and 1.9 Å^–1^ which correspond to *d*-spacings of 12.45 and 3.29 Å, respectively. The first peak can be interpreted as standing molecules of EPI with a slight tilt of the long molecular axis away from the substrate normal (EPI has a molecular length of ∼12.6 Å).^5^ The second peak gives a *d*-spacing similar to the intermolecular distance of π–π interactions, suggesting that molecules lying parallel to the substrate surface are also present. The presence of molecules with two distinct orientations in the same film is not uncommon^38–41^ and the broad, weak nature of the diffraction peaks observed for films prepared at 200 K shows that the film is not well ordered.^42^ The fact that films produced at 200 K are mostly disordered can potentially explain, and is consistent with, the observed poor device performance of EPI transistors prepared at substrate temperatures of 200 K,^5^ as the degree of molecular ordering is strongly correlated with charge transport mobility in thin films.^43^

Films prepared at 300 K are much better ordered (seen from the increased intensity and sharpness of diffraction peaks) and several Bragg peaks are visible at 0.49, 0.57, 0.99 and 1.99 Å^–1^, which correspond to *d*-spacings of 12.66, 11.11, 6.34 and 3.15 Å, respectively. The peak at 0.49 Å^–1^ suggests a structure containing upright-standing molecules, similar to films prepared at 200 K, however now with a reduced tilt with respect to the surface normal; the peak at 0.99 Å^–1^ is the second order reflection of the same peak series. The subtle change in molecular tilt with respect to the structure observed in films prepared at 200 K suggests that there are potentially several polymorphs of EPI which differ primarily in the tilt of the long molecular axes (something previously observed for other rod-like molecules).^9^ The peak at 0.57 Å^–1^ suggests a second, more tilted polymorph of EPI is also present, with the peak having a reduced intensity compared with that of the other polymorph. The *d*-spacing of 11.02 Å to which it corresponds, again suggests molecules with an approximately upright-standing orientation, but now with an increased tilt away from the surface normal. This scenario is similar to the polymorphic behavior of the prototypical organic semiconductor pentacene, where several different polymorphs are known and can coexist within a thin film, each phase with a different out-of-plane *d*-spacing corresponding to a different molecular tilt.^44^ Therefore, the presence of multiple phases may suggest that a so-called surface-mediated phase^9^ is present in films of EPI. Finally, the comparably weak peak at 1.99 Å^–1^ shows that flat-lying molecules are again found to exist in the film, as this corresponds to the approximate length of a short intermolecular π–π interaction (3.15 Å).^14^ Overall, in EPI films prepared at 300 K, at least two (perhaps three) polymorphs are present with two distinct molecular orientations.

The third EPI film, prepared at a substrate temperature of 350 K, is also better ordered and has three Bragg peaks at 0.51, 1.01 and 2.02 Å^–1^, which correspond to *d*-spacings of 12.43, 6.22 and 3.11 Å, respectively. The first and most intense peak at 0.51 Å^–1^ again shows molecules in an approximately upright-standing orientation and appears to be the same phase which is present in films prepared at 200 K, and very similar, but not identical, to the phases present at 300 K; the second peak at 1.01 Å^–1^ is the second order reflection of the same peak series. The final peak at 2.02 Å^–1^ again suggests an approximately flat-lying molecular orientation and a coexistence of standing and lying domains. In all cases, films show defined textures with preferred orientations with respect to the substrate.

As different structures and domains are observed in relatively thick (∼100 nm) films, EPI films with an approximate thickness of 7 nm were investigated using X-ray reflectivity (XRR) measurements to observe if the structure is different in thinner films where the growth is not allowed to proceed. Such studies are of particular interest for the films prepared at 300 K, as it may allow the observation of which phase forms first and if there may be a thickness dependence for the different phases. The XRR data were fitted using a model-independent fitting routine^20^ to obtain an electron density profile of the film perpendicular to the substrate surface. Details on the morphology of ∼7 nm thick films by AFM are given in Fig. S1 of the ESI.†

**Fig. 3:**
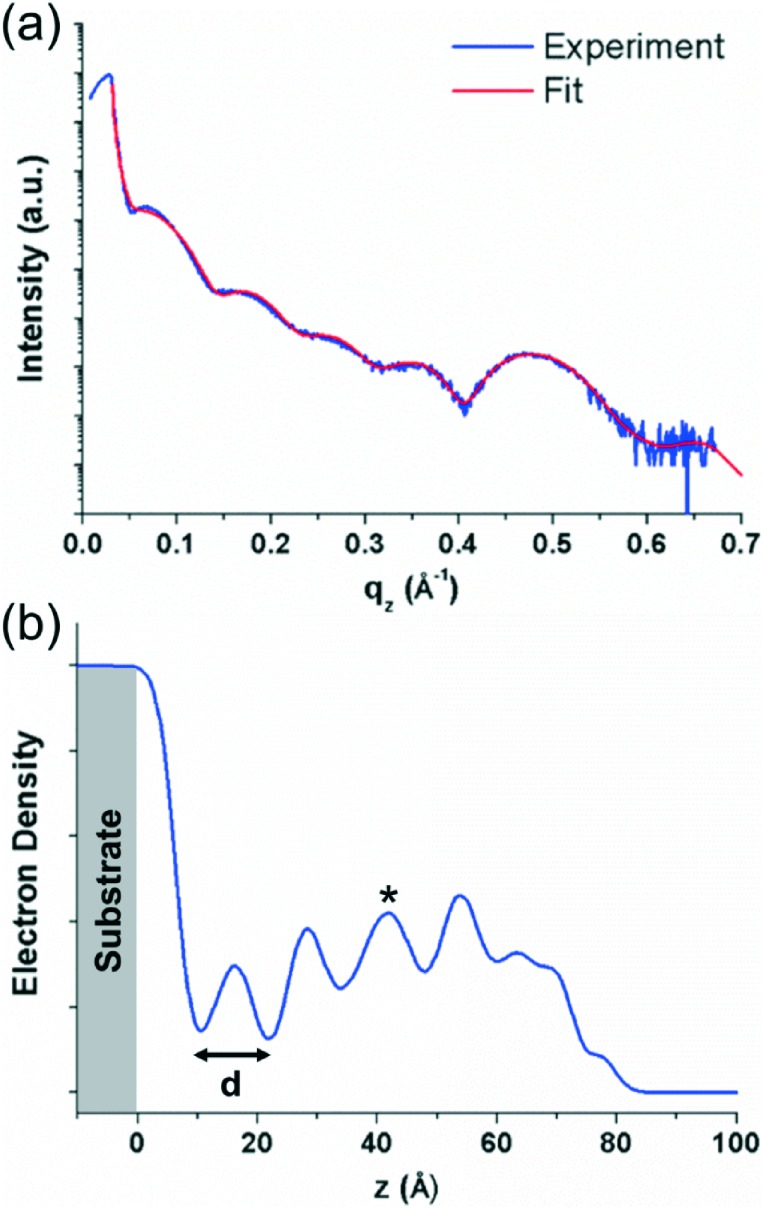
(a) X-ray reflectivity curve (blue) and fit to the data (red) for an ∼7 nm thick epindolidione film grown at a substrate temperature of 300 K. (b) Normalized electron density profile generated from the fit of the X-ray reflectivity data plotted against film depth for the same epindolidione thin film. The substrate is shown colored in grey, *d* represents the approximate thickness of one molecular layer.

The XRR curve and fit of the data for the sample prepared at 300 K are shown in Fig. 3(a). The data show the rapid decrease in intensity above the critical angle of the substrate (∼0.04 Å^–1^ in *q*_*z*_), typical for an XRR curve, followed by Kiessig fringes which give information on the thickness of the organic layer. Finally, at ∼0.47 Å^–1^ in *q*_*z*_, a Bragg peak can be observed which corresponds to the most intense Bragg peak observed in the specular X-ray diffraction measurements of the ∼100 nm thick sample (see Fig. 2). The fit of the data is shown superimposed over the experimental data in red in Fig. 3(a) and the electron density profile generated from the fit is shown in Fig. 3(b). The electron density profile shows regular oscillations which correspond to the periodic nature of the crystal packing perpendicular to the substrate surface, again, indicative of a well ordered film. The lower electron density of the first layer suggests that it is less ordered, and a better ordered film develops as subsequent layers are added. The decreasing electron density at higher *z* values (>60 Å) arises from the surface roughness of the film. The distance, *d*, determined from these oscillations gives a value of ∼12 Å for the thickness of one molecular layer. This again suggests approximately upright-standing molecules, though it is not clear which of the two standing phases observed in the specular X-ray diffraction data is present in the thinner films. The shape of the electron density plot for layers above the first layer suggests that the curve could be the result of a superposition of the electron density profiles for the two different phases; this would also explain the slightly irregular peak shapes (*e.g.* for the third layer, marked by an asterisk in Fig. 3(b)). The influence of the individual phases cannot be ascertained from the electron density plot, however, from the specular diffraction data of the thicker film (Fig. 2), the peak corresponding to the less tilted phase is more intense. The XRR curves of the films prepared at 200 and 350 K do not show the same degree of densely packed periodic ordering perpendicular to the substrate and have a more disordered structure (see Fig. S2 in the ESI†).

**Fig. 4:**
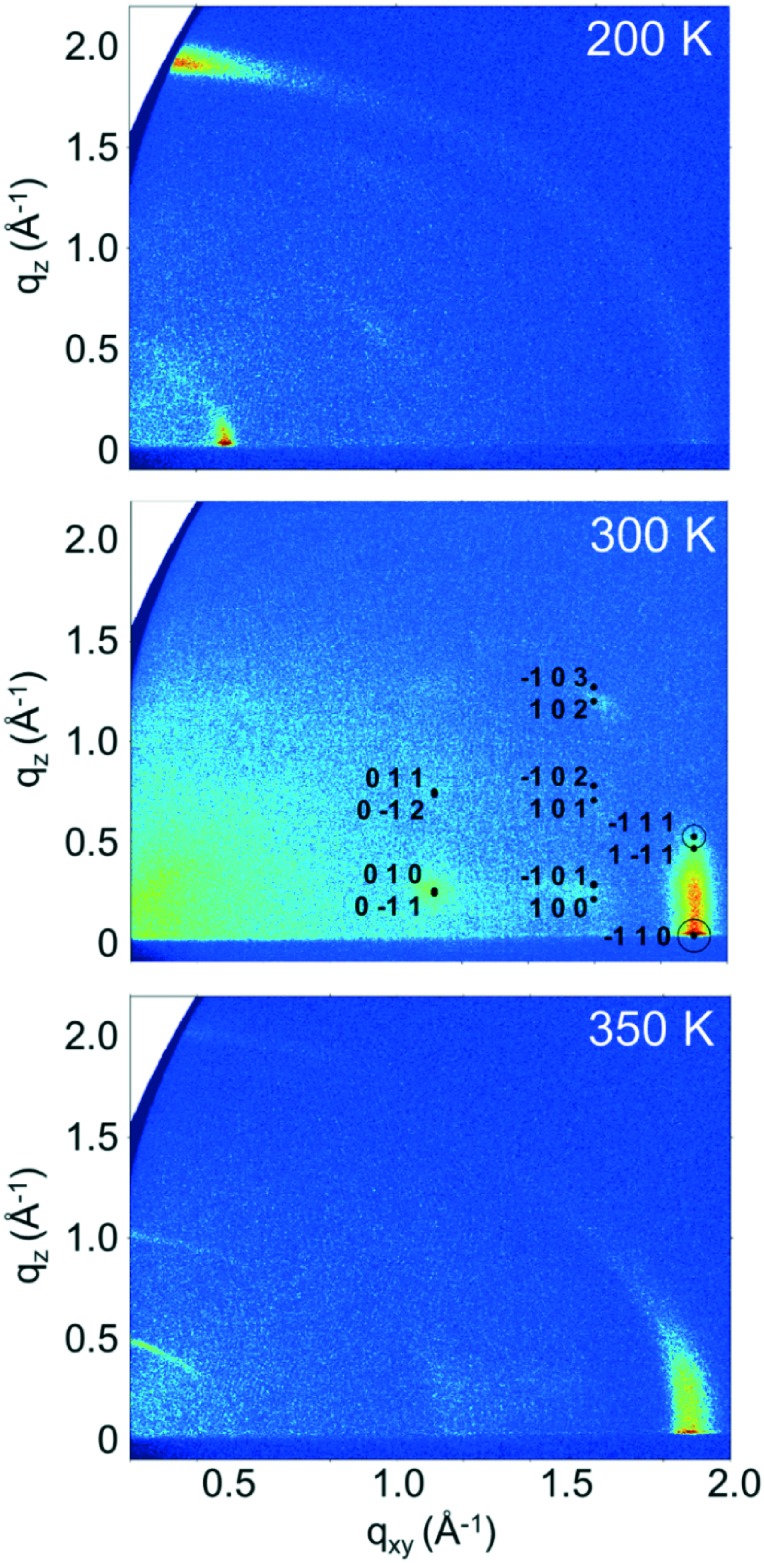
Reciprocal space maps generated from grazing incidence X-ray diffraction data from ∼100 nm thick epindolidione thin films prepared at different substrate temperatures. For the data from the 300 K sample (middle), calculated positions of Bragg peaks from the simulated structure and their respective Miller indices are shown. The areas of the circles around calculated peak positions are proportional to the calculated structure factors of the simulated epindolidione crystal structure.

On determining the presence of several polymorphs and different orientations of EPI molecules in films by analyzing the out-of-plane order within the films, grazing incidence X-ray diffraction (GIXD) measurements were performed to gain insight into the in-plane ordering, which is inaccessible to specular X-ray diffraction measurements. Reciprocal space maps generated from GIXD data for the ∼100 nm thick samples prepared at different substrate temperatures are shown in Fig. 4. The maps show the in-plane scattering vector, *q*_*xy*_, plotted against the out-of-plane scattering vector, *q*_*z*_, and reveal several clear diffraction features for each of the samples.

Starting with the data for the sample prepared with a substrate temperature of 350 K (Fig. 4, bottom), three features are visible at *q*_*z*_ positions of ∼0.5, 1.0 and 2.0 Å^–1^. These peak positions are in good agreement with the peaks observed in the specular diffraction data presented in Fig. 2 (peaks would intercept the *q*_*z*_ axis at ∼0.51, 1.01 and 2.02 Å^–1^). These features extend along Debye–Scherrer rings (rings along a constant *q* value), showing that while there is a preferred orientation of the molecules on the surface, there is also mosaicity present and the texture is not highly defined. The first two stronger features at lower *q* correspond to approximately upright-standing molecules, while the weaker feature at ∼2.0 Å^–1^ corresponds to molecules lying on the substrate surface. A vertical feature resembling a rod smeared along a Debye–Scherrer ring is observed at *q*_*xy*_ ≈ 1.88 Å^–1^, which corresponds to a *d*-spacing of 3.34 Å. This distance agrees well with the length typically associated with intermolecular π–π interactions and is in agreement with the observation of primarily standing molecules, as it is observed in the in-plane direction. The fact that no sharp Bragg peaks are observed, coupled with the presence of a rod-like feature in the in-plane direction points to a disordered layered structure: the film is partially ordered into layers perpendicular to the substrate surface, but there is no inter-layer ordering present as would be observed for a three-dimensionally ordered crystal.

For the sample prepared with a substrate temperature of 200 K (Fig. 4, top), the situation is markedly different. Here, only one strong peak is observed along the direction of the vertical axis at *q*_*z*_ ≈ 1.9 Å^–1^. The peak also extends weakly along a Debye–Scherrer ring, again pointing to significant mosaicity in the film. However, the strength of this feature close to *q*_*xy*_ = 0 shows that there is a strong preferred orientation, with a significant amount of lying molecules at the surface, as this peak corresponds to a *d*-spacing of 3.3 Å which can be related to intermolecular π–π interactions. The second significant feature is an in-plane peak at *q*_*xy*_ ≈ 0.49 Å^–1^, which corresponds to a *d*-spacing of 12.9 Å, approximately the length of an EPI molecule. This feature confirms molecules lying with their long molecular axes parallel to substrate surface, and is in contrast to the situation observed in films prepared with a substrate temperature of 350 K, where molecules are mostly approximately upright-standing. The fact that the peak corresponding to the presence of lying molecule in the specular scan at *q*_*z*_ = 1.9 Å^–1^ (Fig. 2) is not stronger when compared with the samples prepared at 300 and 350 K can be explained by the significant mosaicity of the 200 K film seen in the GIXD measurement (Fig. 4). This result shows that it is possible to tune the molecular orientation of the molecules on the surface only by changing the substrate temperature during deposition. The observed orientation of molecules in films prepared at a substrate temperature of 200 K also now clearly explains the observed poor performance of EPI transistors prepared in this manner,^5^ as charge transport through the film parallel to substrate is less favored due to the lying molecular orientation (*i.e.*, perpendicular to the direction of the intermolecular π–π interactions). The absence of sharp Bragg peaks, and the presence of features resembling rods, again points to a disordered layered structure and highlights the poor order within the films, in agreement with the specular X-ray diffraction and XRR data.

The final sample, prepared at a substrate temperature of 300 K (Fig. 4, middle), again shows a different behavior. The peaks observed in the specular diffraction pattern (Fig. 2) are not observed here (regions close to *q*_*xy*_ = 0 cannot be observed in GIXD reciprocal space maps), suggesting reduced film mosaicity (or a stronger preferred orientation) compared with the other samples. A strong rod-like feature is present at *q*_*xy*_ ≈ 1.9 Å^–1^ (*d* = 3.3 Å), showing evidence of a disordered layer structure with molecules approximately upright-standing on the substrate surface, similar to the 350 K sample. However, in the case of the samples prepared at 300 K, Bragg peaks can also be observed at *q*_*xy*_ values of ∼1.1 and 1.6 Å^–1^ and various *q*_*z*_. These Bragg peaks show that a three-dimensionally ordered species is also present with a preferred orientation on the substrate. Using the unit cell parameters from the previously determined structures of the 2,8-disubstituted chlorinated and fluorinated EPI derivatives (2F-EPI and 2Cl-EPI; CSD codes DUXYIG and DUXYOM, respectively)^11^ as a starting point (chemical structures are given in Fig. S3 of the ESI†), the Bragg peaks can be indexed and the unit cell parameters for EPI in thin films can be determined. The unit cell is given in Table 1, with EPI adopting a 001 orientation (the 001 lattice plane parallel to the substrate) in the 300 K thin film. This orientation of the unit cell means that EPI molecules are approximately upright-standing on the substrate surface, in agreement with the specular X-ray diffraction and XRR data.

**Table 1:** Unit cell parameters of the structure of epindolidione observed in films prepared with a substrate temperature of 300 K in comparison with the previously determined structures of the fluorinated (2F-EPI) and chlorinated (2Cl-EPI) derivatives^11^

Compound	*a* (Å)	*b* (Å)	*c* (Å)	*α* (°)	*β* (°)	*γ* (°)	V (Å^3^)
EPI	3.94	5.64	13.09	101.70	96.75	93.00	282.0
2F-EPI	3.690(1)	5.976(1)	13.489(2)	101.785(2)	94.892(6)	92.309(10)	289.61(10)
2Cl-EPI	3.900(3)	6.260(5)	13.593(12)	90.947(11)	96.272(10)	98.599(9)	326.0(5)

**Fig. 5:**
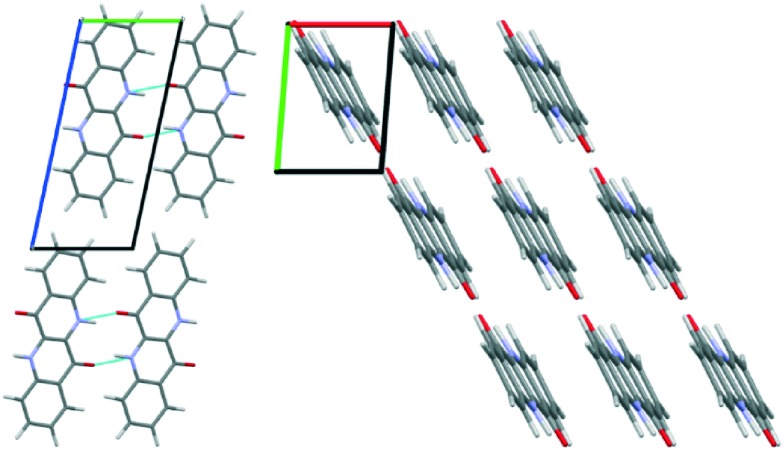
Crystal packing of epindolidione molecules in films prepared at substrate temperatures of 300 K: viewed along the *a*-axis showing the interlayer packing and hydrogen bonding pattern (left) and viewed along the *c*-axis showing the intermolecular π–π interactions (right). The unit cell dimensions are shown in both images.

The unit cell parameters determined from the 300 K film show a triclinic structure and are very similar to those determined for 2F-EPI, suggesting a similar type of packing motif. The volume of the unit cell means that only one EPI molecule is present in the unit cell (*Z* = 1). On the basis of this information, molecular dynamics (MD) simulations were performed to get information regarding the orientation of the molecule within the unit cell and to obtain a crystal structure solution for EPI in the 300 K thin films. Initial structures were generated by expanding the experimentally determined unit cell volume by 20% and randomly placing one molecule within the cell. MD simulation runs over 70 ps were performed, during which the unit cell was shrunk back to the experimentally determined dimensions while the systems relaxed. The resulting structures were first ordered based on energy, before several hundred of the lowest energy structures were grouped based on similarity according to certain criteria (*e.g.* Hausdorff distance to the lowest energy structure, π–π interaction distance, *etc.*). A representative structure from each group was then further optimized using dispersion corrected density functional theory (DFT) calculations. The simulated diffraction patterns of these structures were then compared to the experimental diffraction pattern and the final structure was chosen based on a comparison of the experimentally determined and simulated structure factors (Fig. 4, middle); this structure was also the lowest energy structure obtained from the DFT optimizations. Please note that the accuracy of this crystal structure is restricted based on experimental limitations (*e.g.* certain corrections to the peak intensities could not be applied due to the broad and weak nature of the observed Bragg peaks) and also the sample quality (only a small number of low intensity peaks are observed). However, the fact that a previously unknown crystal structure of a material can be determined from a thin film diffraction pattern based on a very limited dataset (∼7 Bragg peaks) is worth highlighting. The final structure determined from the thin film by the MD simulations and DFT optimization is shown in Fig. 5. The structure has a layered motif of molecules hydrogen-bonded to neighbors *via* two N–H···O hydrogen bonds, with N···O distances of 2.68 Å (Fig. 5, left), and intermolecular π–π interactions with neighboring molecules with a distance of 3.47 Å (Fig. 5, right). The hydrogen bond distance found in the structure is relatively short, but not uncommon.^45^ It should also be noted that this distance is likely strongly dependent on the choice of unit cell parameters; as there is only one molecule in the unit cell, it only self-interacts with molecules in adjacent unit cells.

A final confirmation that the determined structure is plausible can be made by comparison with the known fluorinated and chlorinated derivative crystal structures (2F-EPI and 2Cl-EPI, respectively).^11^ A first visual comparison can be made by overlaying the conjugated backbone of a molecule in the EPI structure with those from the derivative structures and then generating the surrounding molecules of both structures to observe if there is an agreement in the extended crystal packing (see Fig. S4 in the ESI†). From this analysis, it can be seen that there is an excellent agreement between the packing in the EPI structure and that of the fluorinated derivative (2F-EPI), this is an encouraging indicator with regards to the chosen EPI structure. The similarity in the structures is perhaps unsurprising given the similar unit cell parameters (there is only a small reduction in volume of the EPI unit cell compared with the fluorinated derivative, most likely arising from the replacement of fluorine atoms with smaller hydrogen atoms). It should be noted here that the structures of the derivatives were not used as an input or reference at any point during the MD and DFT simulations, and were only studied once a final structure for EPI in the 300 K thin film had been chosen.

**Fig. 6:**
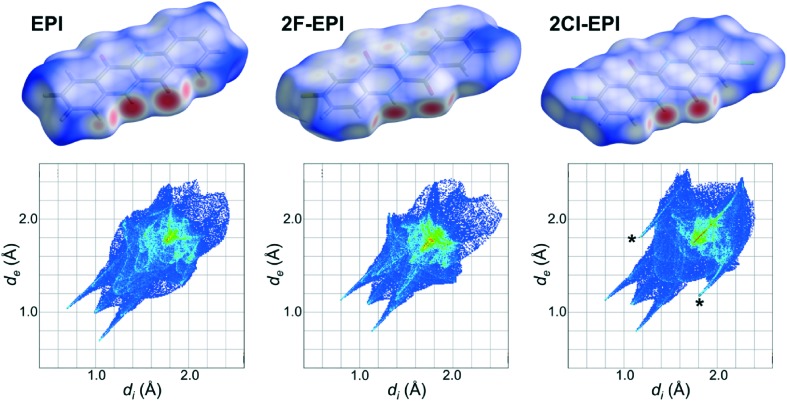
Hirshfeld surfaces for the crystal structure of epindolidione (EPI) solved from a thin film and the fluorinated and chlorinated derivatives (2F-EPI and 2Cl-EPI),^11^ with their corresponding fingerprint plots shown below. Hirshfeld surfaces are mapped over a *d*_norm_ range of –0.6 to 1.2 Å, while fingerprint plots are plotted as *d*_i_ *vs. d*_e_ in Å.

A more quantitative comparison of the crystal structures and the intermolecular interactions taking place can be obtained using Hirshfeld surfaces and their corresponding fingerprint plots. An explanation of the generation of Hirshfeld surfaces is given in the experimental section and in the literature citations.^34–36^ Hirshfeld surfaces for EPI and the two halogenated derivatives, 2F-EPI and 2Cl-EPI, are shown in Fig. 6 mapped over a *d*_norm_ range of –0.6 to 1.2 Å. The strongest intermolecular interactions between neighboring molecules, shown as the red areas on the Hirshfeld surfaces, occur for the N–H···O hydrogen bonds, blue areas correspond to areas with no close contacts (shorter than the sum of the van der Waals radii). Comparing the fingerprint plots of the different structures, again confirms the similarity of the EPI structure with that of 2F-EPI. The main difference between the fingerprint plot of the chlorinated derivative and those of the other structures is the presence of two wings, marked by asterisks in Fig. 6, at (*d*_i_, *d*_e_) positions of (1.8 Å, 1.2 Å) and (1.2 Å, 1.8 Å). These arise from C–H···Cl interactions, which are clearly absent in the other two structures. For the 2F-EPI structure, the analagous C–H···F interactions occur over similar (*d*_i_, *d*_e_) positions to the H···H interactions, showing that the addition of larger chlorine atoms to the conjugated backbone disrupts the packing of the hydrogenated EPI structure more than the addition of smaller fluorine atoms. A quantitative analysis of the contributions of different interactions to the Hirshfeld surface area shows that, for EPI and 2F-EPI, the H···H interactions are dominant, with contributions to the Hirshfeld surface area of 42.7% (EPI) and 20.5% (2F-EPI). For all three structures, contributions are also made by the O···H interactions arising from the hydrogen bonds (15.9% for EPI, 16.3% for 2F-EPI and 14.9% for 2Cl-EPI). In the case of 2Cl-EPI, the dominant interactions are Cl···H (23.6%), closely followed by H···H interactions (18.8%), while for 2F-EPI a significant contribution is also made by F···H interactions (18.8%). Generally, the Hirshfeld surfaces show the slight dominance of interactions involving the substituents to the conjugated backbone (involving H, F and Cl), as opposed to π interactions, C···C (mostly π–π) and C···H (mostly C–H···π) interactions (30.2% in EPI, 27.2% in 2F-EPI and 25.8% in 2Cl-EPI). A graphical representation of the relative contributions of different interactions to the Hirshfeld surfaces is given in the ESI† (Fig. S5).

**Fig. 7:**
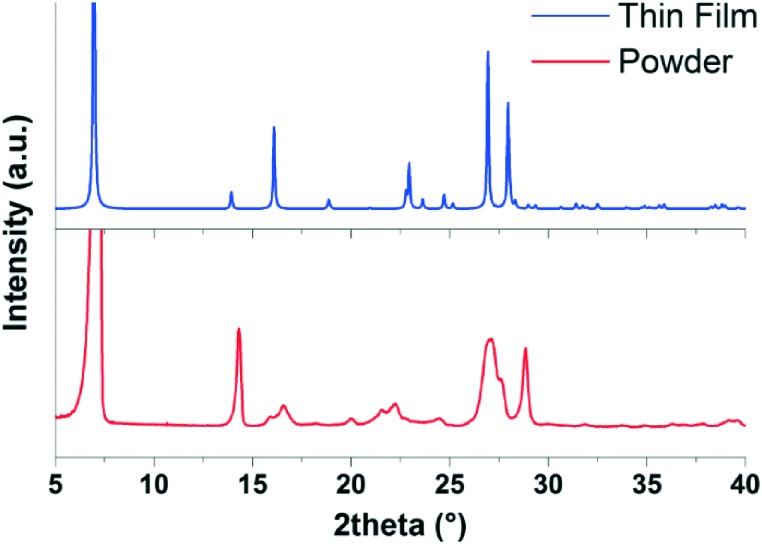
Comparison of the diffraction pattern of the bulk epindolidione (EPI) powder (red) with the simulated powder diffraction pattern of the structure solved from the 300 K EPI thin film (blue).

The final comparison which can be made is of the simulated powder diffraction pattern from the structure solved from the 300 K thin film with the experimentally determined powder diffraction pattern from the bulk powder used for the deposition of the films. As it is known that new polymorphs can form when a material crystallizes at a substrate surface, this is an important check to see if the structure obtained here is the bulk polymorph or a surface-mediated polymorph.^9^ The diffraction patterns of the bulk EPI powder and the simulated powder diffraction pattern of the structure solved from the thin film are shown in Fig. 7. There is a clear difference in the two powder diffraction patterns, suggesting that the structure solved from the thin film is actually a surface-mediated polymorph and not the bulk structure present in the powder. It should be noted, that it was not possible to index the diffraction pattern of the bulk EPI powder due to the significant anisotropic peak broadening caused by crystal defects and particle size effects (compare, for example, peaks shapes of the bulk powder with the calculated diffraction pattern of the thin film polymorph in Fig. 7). Therefore, from the data presented here, it is not possible to offer a crystal structure solution for the bulk EPI polymorph. As previously stated, it has been observed that growth of large, defect free crystals of EPI and the closely related family of quinacridone hydrogen-bonded pigments is challenging;^11,12^ this is also the likely reason that a crystal structure for EPI has not, until now, been solved. As no known bulk unit cell parameters are available, a direct comparison between the thin film diffraction pattern (see Fig. 4) and the bulk structure cannot be made. However, from visual comparison of the bulk EPI powder diffraction pattern and the simulated powder diffraction pattern of the structure solved from the thin film, it is clear that a different polymorph is present, adding a further level of complexity to the crystallization behavior of epindolidiones. The presence of a surface-mediated polymorph of EPI is significant, as it is likely this structure, and not the bulk polymorph, which dictates the properties of the high stability OFETs and organic light-emitting diodes (OLEDs) previously produced using this material.^11^

## Conclusions

In summary, thin films of EPI, with thicknesses of either ∼100 nm or ∼7 nm, deposited at three different substrate temperatures have been structurally characterized. While the film morphologies are relatively similar, different molecular orientations and tilts with respect to the substrate surface are found depending on the substrate temperature used during deposition; molecules are mostly approximately upright-standing in films prepared at 300 and 350 K, while films prepared at 200 K are mostly comprised of lying molecules. In the samples prepared at 300 K, where two approximately upright-standing phases are present, XRR measurements on very thin films (∼7 nm) could not distinguish which phase forms first during early film growth and it is suggested both phases grow at the same time. Films prepared with substrate temperatures of 200 and 350 K do not show a high degree of order, and are mainly comprised of disordered layered structures, with only out-of-plane order and very little in-plane order. An exception is observed for the film deposited at a substrate temperature of 300 K, where several Bragg peaks are observed in the GIXD data which could be indexed to provide a unit cell for EPI in the thin film. From this, the crystal structure in the thin film was solved using MD simulations and DFT optimization, followed by comparison of the structure factors of the most promising simulated structures with the experimental data. As the method does not rely on the evaluation of diffraction peak intensities (the crystal packing is derived from an energetic point of view), it allows for a crystal structure to be determined for systems where only a small number of weak, diffuse Bragg peaks are observed, such as in the case of EPI presented here. A crystal structure similar to the previously determined structure of the fluorinated EPI derivative was found.^11^ An assessment of the different interactions occurring within the crystal structures of EPI and the halogenated derivatives further emphasizes the similarity of the thin film structure to that of the fluorinated derivative, as opposed to the chlorinated derivative. Comparison of the simulated powder diffraction pattern from the crystal structure solved from the thin film with the diffraction pattern of the bulk EPI powder used for the deposition shows some clear differences, suggesting the structure found in the thin film is in fact a surface-mediated polymorph. It is therefore likely that this polymorph is responsible for the performance of some of the organic devices previously produced using this material,^11^ while the observation of lying molecules in films produced at 200 K explains the poor performance of OFETs produced from films deposited in such a way.^5^
