# Supplementary material for: Solution of an elusive pigment crystal structure from a thin film: a combined X-ray diffraction and computational study
Source: CrystEngComm. 2017 Mar 14;19(14):1902–11. doi: 10.1039/c7ce00227k (PMC5436089; doi:10.1039/c7ce00227k)
Supplement: Supplementary file 1 [file CE-019-C7CE00227K-s001.pdf]

Electronic Supplementary Material (ESI) for CrystEngComm.
This journal is © The Royal Society of Chemistry 2017

Electronic Supplementary Information for:

Solution of an Elusive Pigment Crystal Structure from a Thin Film: a Combined X-ray Diffraction and Computational Study

Andrew O. F. Jones,[ab*] Christian Röthel,[ac] Roman Lassnig,[a] O. N. Bedoya-Martínez,[a] Paul Christian,[a] Ingo Salzmann,[d] Birgit Kunert,[a] Adolf Winkler,[a] and Roland Resel,[ab]

[a] Institute of Solid State Physics, Graz University of Technology, Petersgasse 16, 8010 Graz, Austria

[b] BioTechMed-Graz, Austria

[c] Institute for Pharmaceutical Sciences, Department of Pharmaceutical Technology, Karl-Franzens University of Graz, Universitätsplatz 1, 8010 Graz, Austria

[d] Institut für Physik, Humboldt-Universität zu Berlin, Brook-Taylor Straße 6, 12489 Berlin, Germany

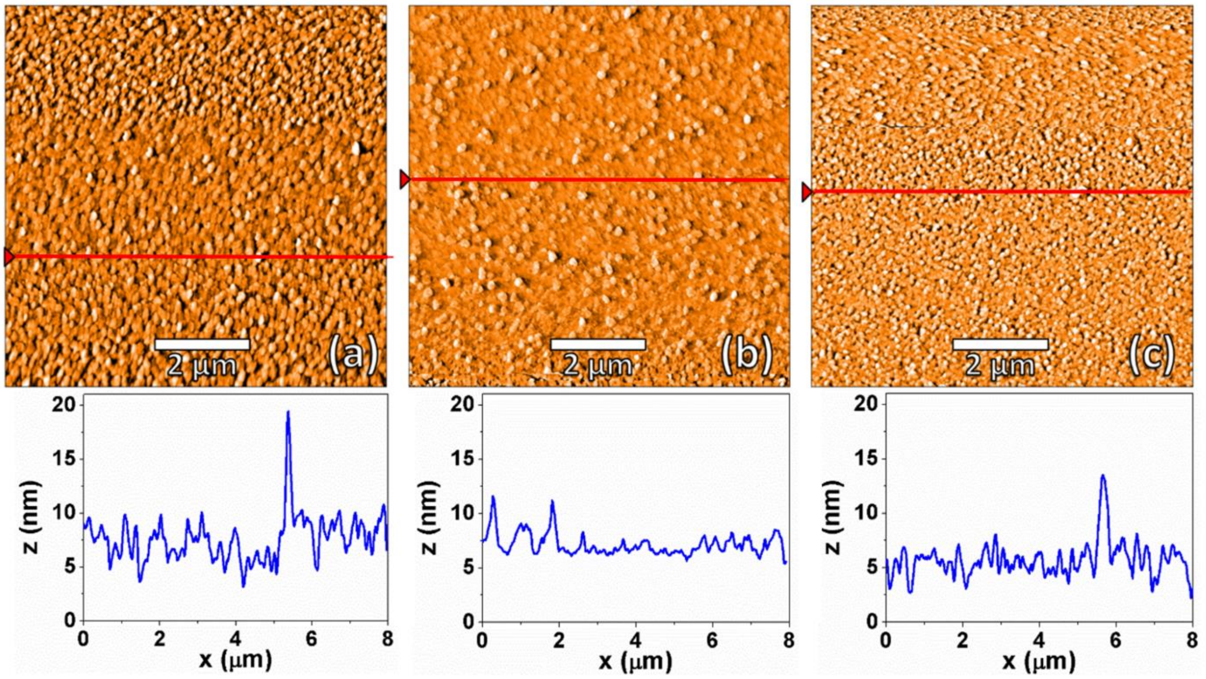

Fig. S1 AFM images (top) of epindolidione thin films with a nominal thickness of ~7 nm grown on SiO_2 surfaces at substrate temperatures of 200 K (a), 300 K (b) and 350 K (c). The red lines

correspond to the areas from which line profiles (bottom) have been generated where the zero value for z has been set to the lowest point measured in the AFM image.

Morphologies of the ~7 nm thick films (Fig. S1) are similar to those observed for thicker EPI films (Fig. 1). In this case, larger domains are observed for samples at a lower temperature during deposition, while there is not a significant difference in the film roughnesses.

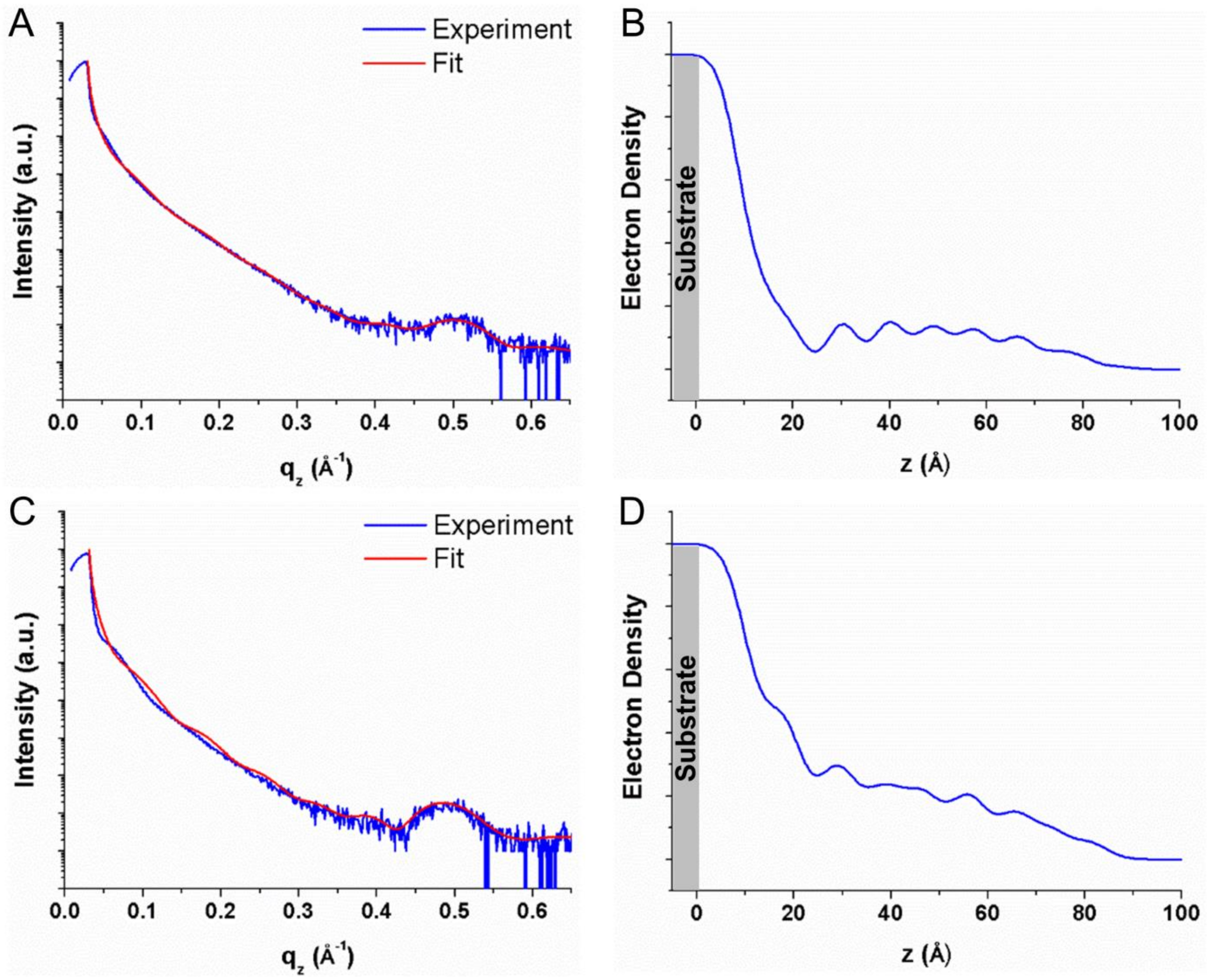

Fig. S2 A and C: X-ray reflectivity curves (blue) and fits to the data (red) from epindolidione films grown at substrate temperatures of 200 and 350 K, respectively. B and D: Normalized electron density profiles generated from the model fits of the X-ray reflectivity data from epindolidione films grown at substrate temperatures of 200 and 350 K, respectively. The substrate is shown colored in grey.

The model independent fits of the XRR data (Fig. S2, A and C) show good agreement with the experimental data. The electron density profiles of the films (Fig. S2, B and D), perpendicular to the substrate surface, generated from the fits, do not show any periodic shape for the sample prepared at a substrate temperature of 350 K, while some ordering is seen in the 200 K sample. However, the packing is much less dense in the 200 K sample compared with both the 300 and 350 K samples. This shows that the films are not as well ordered into layers perpendicular to the substrate surface and the films are relatively disordered in comparison with the films prepared at substrate temperatures of 300 K.

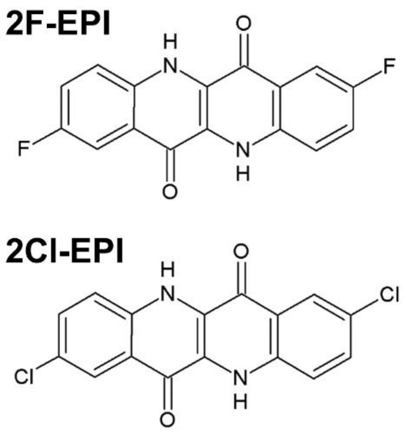

Fig. S3 Chemical structure diagrams of the halogenated EPI derivatives, 2F-EPI (top) and 2Cl-EPI (bottom).

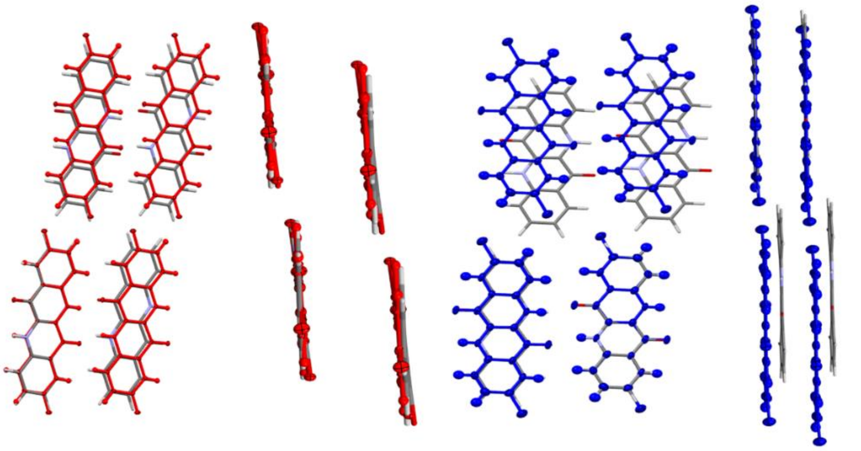

Fig. S4 Structural overlays of the simulated structure of EPI (grey) with 2F-EPI (red, left) and 2Cl-EPI (blue, right),[1] each viewed in two orientations. In each image, the backbone of one EPI molecule is overlayed with the one from the other compared structure (2F-EPI or 2Cl-EPI), before generating the surrounding molecules to compare the general packing motif.

Fig. S4 shows the comparison of the simulated structure of EPI with those of the fluorinated and chlorinated derivatives. The packing in the structures of EPI and 2F-EPI are found to be very similar.

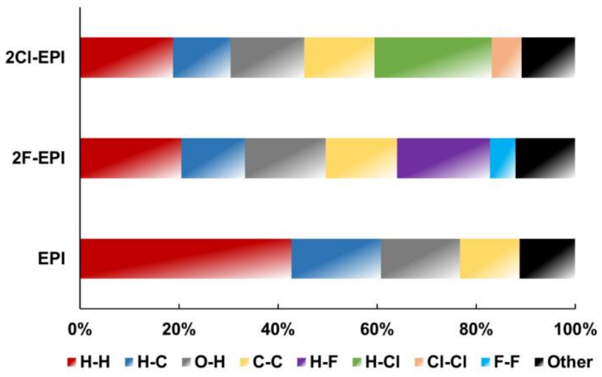

Fig. S5 Graphical representation of the contributions of different interactions to the Hirshfeld surfaces presented in Fig. 6 for EPI, 2F-EPI and 2Cl-EPI.

References

1 E. D. Głowacki, G. Romanazzi, C. Yumusak, H. Coskun, U. Monkowius, G. Voss, M. Burian, R. T. Lechner, N. Demitri, G. J. Redhammer, N. Sünger, G. P. Suranna and N. S. Sariciftci, *Adv. Funct. Mater.*, 2015, **25**, 776–787.
